# Synthesis and Characterization of Alkali Metal Ion-Binding Copolymers Bearing Dibenzo-24-crown-8 Ether Moieties

**DOI:** 10.3390/polym10101095

**Published:** 2018-10-02

**Authors:** Da-Ming Wang, Yuji Aso, Hitomi Ohara, Tomonari Tanaka

**Affiliations:** Department of Biobased Materials Science, Graduate School of Science and Technology, Kyoto Institute of Technology, Kyoto 606-8585, Japan; d6861002@edu.kit.ac.jp (D.M.W.); aso@kit.ac.jp (Y.A.); ohara@kit.ac.jp (H.O.)

**Keywords:** crown ether, radical polymerization, molecular recognition, alkali metal, cesium

## Abstract

Dibenzo-24-crown-8 (DB24C8)-bearing copolymers were synthesized by radical copolymerization using a DB24C8-carrying acrylamide derivative and *N*-isopropylacrylamide monomers. The cloud point of the resulting copolymers changed in aqueous solution in the presence of cesium ions. In addition, the ^1^H NMR signals of DB24C8-bearing copolymers shifted in the presence of alkali metal. This shift was more pronounced following the addition of Cs^+^ compared to Rb^+^, K^+^, Na^+^, and Li^+^ ions due to recognition of the Cs^+^ ion by DB24C8.

## 1. Introduction

The general population is exposed to few cesium compounds, which are mildly toxic due to the chemical similarity of cesium and potassium [1,2]. Radiocesium is a common component of nuclear fission products. Radioactive waste treatment gained importance following the crisis at the Fukushima Daiichi Nuclear Power Plant in Japan in 2011. In particular, the radiocesium isotopes ^134^Cs and ^137^Cs, which have half-lives of 2.1 years and 30.2 years, respectively, pose significant long-term human health concerns [3], but the development of efficient and selective reagents for adsorbing cesium from aqueous environments remains challenging [4].

Crown ethers are macrocyclic compounds with the unique property of binding specific metal ions in their cavity, depending on the size of the cavity [5,6]. For example, large 24-crown-8 ethers can bind specific metal ions, including cesium [7,8,9,10,11,12,13], and are used to extract cesium from aqueous environments [14], to form cesium ion-imprinted polymer nanoparticles [2], and to adsorb and immobilize cesium in inorganic silica material [15]. We therefore predicted that the incorporation of a 24-crown-8 ether into a synthetic polymer backbone would provide a useful material capable of the molecular recognition of specific metal ions such as cesium.

Poly (*N*-isopropylacrylamide) (PNIPAM) is well-known as a temperature-responsive polymer and has a cloud point in water of around 32 °C. Hydrogen bonds form between water and the amide groups below the cloud point, making PNIPAM solutions transparent. In contrast, when the solution temperature is above the cloud point, the hydrogen bonds collapse and an aggregation forms. The incorporation of hydrophilic or hydrophobic co-monomers allows the cloud point of copolymers to be tuned. The attractive and tunable properties of PNIPAM has resulted in the synthesis of many copolymers and hydrogels using PNIPAM which are used in various applications such as drug delivery and recyclable absorbents [16,17,18,19,20,21,22,23].

Several PNIPAM linear copolymers containing benzo-18-crown-6 [17,24,25], benzo-12-crown-4 [25], benzo-15-crown-5 [25], and dibenzo-18-crown-6 monomers [26] have been, reported and exhibit both temperature-responsive and ion-recognition properties (i.e., their cloud points change in response to specific metal ions). These reports provide valuable information for designing and applying crown ether-based ion-responsive materials to various applications. For example, a selective and efficient methodology is required for removing metal ions from aqueous environments. To our knowledge, there has been no report of PNIPAM modified with the hydrophobic dibenzo-24-crown-8 (DB24C8) monomer. DB24C8 can recognize metal ions, including cesium, and thus the incorporation of DB24C8 into PNIPAM may provide a copolymer that is responsive to Cs^+^. In this paper we describe the synthesis of a new class of copolymers consisting of PNIPAM and DB24C8 using radical copolymerization with a DB24C8-carrying monomer. The properties of the resulting copolymers were investigated in the presence of various metal ions, including Cs^+^.

## 2. Materials and Methods

### 2.1. Materials

*N*-Isopropylacrylamide (NIPAM) was purchased from Wako Pure Chemical Industries (Osaka, Japan) and used after recrystallization from *n*-hexane. Acrylamide (AAM) and 2,2′-azobis(isobutyronitrile) (AIBN) were purchased from Nacalai Tesque Inc. (Kyoto, Japan). 2-Carboxydibenzo-24-crown-8 (**1**) was synthesized according to the reported procedure [27,28,29]. All other reagents were commercially available and used without further purification.

### 2.2. Measurements

^1^H NMR spectra were recorded on a Bruker AVANCE 300 MHz or AVANCE II 600 MHz and the spectra were recorded at 300 K. Mass spectra were taken on a Bruker microTOF or amazon SL mass spectrometer. IR spectra were recorded on a JASCO FT/IR-4600 spectrometer. Gel permeation chromatography (GPC) measurements were conducted using a system consisting of a JASCO PU-2089 pump, a CO-2065 column oven, an RI-2031 refractive index detector, and a Shodex KD-804 (8.0 mm × 300 mm) column. DMF containing 10 mM LiBr was used as the eluent at a flow rate of 0.5 mL min^−1^ at 50 °C. Poly(methyl methacrylate) samples were used as standards. Transmittance was recorded using a JASCO V-550 UV-vis spectrometer. A 0.5 wt % polymer aqueous solution was filtered through a membrane filter (0.45 µm), then the transmittance of the sample solution was measured in a quartz cell (cell length: 10 mm) at 500 nm while heating at a rate of 0.5 °C min^−1^.

### 2.3. Synthesis of DB24C8 Monomer

#### *N*-(2-Carboxydibenzo-24-crown-8)-succinimide (**2**) [28,29]

To a stirred solution of 2-carboxydibenzo-24-crown-8 (**1**) (478 mg, 0.970 mmol) and *N*-hydroxysuccinimide (113 mg, 0.980 mmol) in DMF (20 mL) was added dicyclohexylcarbodiimide (263 mg, 1.27 mmol). After addition, the reaction mixture was stirred at room temperature for 48 h. The solution was filtered by suction filtration to filter off white precipitate and the filtrate was evaporated in vacuo. The crude product was purified by silica gel column chromatography (MeOH:CH_2_Cl_2_ = 1:19) and recrystallized with isopropanol to afford compound **2** as a white solid (443.6 mg, yield 82%). *R*_f_ 0.2 (MeOH:CH_2_Cl_2_ = 1:19); ^1^H NMR (300 MHz, CDCl_3_) δ 7.78 (dd, *J* = 8.5, 1.9 Hz, 1H), 7.55 (d, *J* = 1.9 Hz, 1H), 6.91–6.85 (m, 5H), 4.23–4.14 (m, 8H), 3.97–3.83 (m, 16H), 2.90 (s, 4H); ^13^C NMR (75 MHz, CDCl_3_) δ 169.4, 161.5, 154.6, 148.9, 148.6, 125.6, 121.44, 121.39, 117.2, 114.7, 114.0, 112.0, 71.6, 71.4, 71.3, 70.0, 69.6, 69.5, 69.4, 69.3, (some carbons are hidden into these peaks) 25.7; ESI-MS: *m*/*z* 612.30 (M + Na)^+^, calcd. for C_29_H_35_NO_12_Na: 612.21.

#### 2-[(2-Aminoethyl) carbamoyl]dibenzo-24-crown-8 (**3**)

*N*-(2-Carboxydibenzo-24-crown-8) succinimide (586 mg, 0.990 mmol) in CH_3_CN (12 mL) was added to ethylene diamine (1.20 g, 20.0 mmol) in CH_3_CN (8 mL) solution. After addition, the mixture was stirred at room temperature for 2 h. Then the solution was filtered to remove precipitate and washed with CH_3_CN. The filtrate was evaporated in vacuo to afford crude compound **3** as a pale yellow solid without further purification.

#### 2-(1,6-Dioxo-2,5-diaza-7-oxamyl)dibenzo-24-crown-8 (**4**)

Acryloyl chloride (0.15 mL, 1.84 mmol) was added to a mixture of crude compound **3** and triethylamine (0.27 mL, 1.94 mmol) in CH_2_Cl_2_ (6 mL) solution at 0 °C. After addition, the resulting mixture was stirred at 0 °C for 1 h, and then stirred at room temperature for 19 h. The reaction was quenched by saturated sodium bicarbonate solution (6 mL) and diluted with CH_2_Cl_2_. After extraction, the water layer was re-extracted with CH_2_Cl_2_. The combined organic layer was washed with saturated NaCl aqueous solution, dried over MgSO_4_, filtered, and evaporated in vacuo. The crude product was purified by silica gel column chromatography (MeOH:CH_2_Cl_2_ = 1:19) to afford compound **4** as a white solid (396.7 mg, two steps yield 68%). *R*_f_ 0.15 (MeOH:CH_2_Cl_2_ = 1:9); IR (KBr) 3294, 3082, 2931, 2873, 1658, 1629, 1602, 1594, 1582, 1543, 1507 cm^−1^; ^1^H NMR (600 MHz, CDCl_3_) δ 7.42 (s, 1H), 7.39 (d, *J* = 1.8 Hz, 1H), 7.35 (dd, *J* = 8.3, 1.8 Hz, 1H), 6.93 (s, 1H), 6.89–6.85 (m, 4H), 6.82 (d, *J* = 8.3 Hz, 1H), 6.26 (dd, *J* = 17.0, 1.0 Hz, 1H), 6.14 (dd, *J* = 17.0, 10.3 Hz, 1H), 5.62 (dd, *J* = 10.3, 1.0 Hz, 1H), 4.19–4.13 (m, 8H), 3.92–3.90 (m, 8H), 3.83–3.82 (m, 8H), 3.54 (s, 4H) (Figure S1 (Supplementary Materials)); ^13^C NMR (150 MHz, CDCl_3_) δ 168.0, 167.1, 151.7, 148.9, 148.5, 130.7, 126.8, 126.6, 121.5, 120.4, 114.2, 112.7, 112.6, 71.3, 71.24, 71.21, 71.20, 69.9, 69.8, 69.7, 69.41, 69.38, 69.34, 69.31, 41.1, 40.0 (Figure S2 (Supplementary Materials)); ESI-MS: *m*/*z* 611.212 (M + Na)^+^, calcd. for C_30_H_40_N_2_O_10_Na: 611.258.

### 2.4. Synthesis of DB24C8-Bearing Copolymers

All copolymers were synthesized by free-radical copolymerization using AIBN as an initiator in dimethyl sulfoxide (DMSO) as a solvent. Total monomer concentration was 1.0 M and the molar ratio of AIBN in the total monomer was 1 mol %. NIPAM, DB24C8 monomer (**4**), and AAM were dissolved in 1.5 mL DMSO in a glass tube. The resulting solution was degassed by at least three freeze–thaw cycles, then the glass tube was filled with nitrogen and heated at 70 °C for 24 h. The products were purified by dialysis (Spectra/Por 7 MWCO 1000, Spectrum Laboratories, Inc., Rancho Dominguez, California, CA, USA) against deionized water and freeze-dried to give copolymers. ^1^H NMR spectra are shown in Figures S3–S11 (Supplementary Materials).

### 2.5. Binding Test of Copolymers with Metal Ions by ^1^H NMR

A DMSO-*d_6_* solution (750 μL) containing **P7** or **P9** (7.5 mg) and a D_2_O solution (100 μL) containing alkali metal chloride (0.0143 mmol of LiCl, CsCl for **P7**, 0.0243 mmol of LiCl, NaCl, KCl, RbCl, CsCl for **P9**) were mixed to form copolymer–metal ion solution in DMSO-*d_6_*:D_2_O = 7.5:1 *v*/*v* and recorded. For the competition test, a DMSO-*d_6_* solution (450 μL) containing **P9** (4.5 mg) and a D_2_O solution (60 μL) containing cesium chloride (0.0146 mmol, 2.5 mg) and other alkali metal chlorides (NaCl or KCl) were mixed and recorded on a Bruker AVANCE II 600 MHz.

## 3. Results and Discussion

### 3.1. Synthesis of DB24C8-Bearing Copolymers

**Scheme 1:**
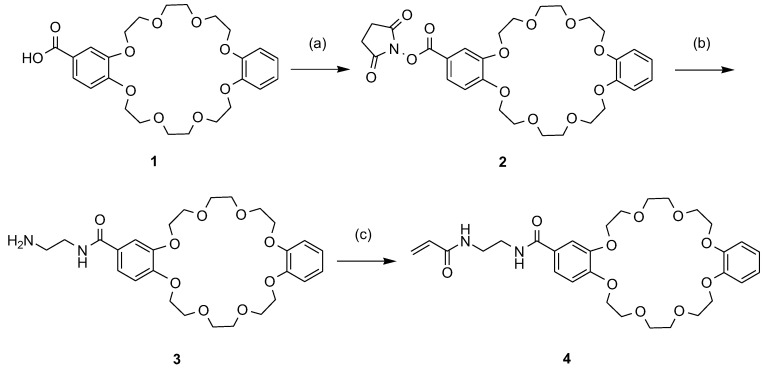
Synthesis of dibenzo-24-crown-8 (DB24C8) monomer. (a): *N*-Hydroxysuccinimide, dicyclohexylcarbodiimide, DMF, r.t., 48 h, 82%; (b): Ethylenediamine, CH_3_CN, r.t., 2 h; (c): Acryloyl chloride, triethylamine, CH_2_Cl_2_, 0 °C–r.t., 20 h, 68% (two steps (b,c)).

**Scheme 2:**
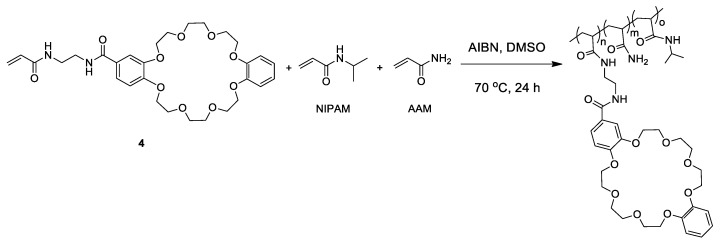
Synthesis of copolymer bearing DB24C8. NIPAM:* N*-isopropylacrylamide; AAM: acrylamide; AIBN: 2,2′-azobis(isobutyronitrile); DMSO: dimethyl sulfoxide.

The DB24C8-carrying AAM derivative was synthesized as outlined in Scheme 1. First, the succinimide ester derivative of DB24C8 (**2**) was obtained from the carboxylic acid-functionalized DB24C8 (**1**) using a literature procedure [29,30]. Next, the activated ester of DB24C8 (**2**) was reacted with excess ethylenediamine to obtain compound **3**, followed by reaction with acryloyl chloride to yield 2-(1,6-dioxo-2,5-diaza-7-oxamyl)dibenzo-24-crown-8 (**4**) as a monomer used for synthesizing the DB24C8-bearing copolymers. A radical copolymerization reaction was carried out using **4**, NIPAM, and AAM as monomers, and AIBN as an initiator (Scheme 2). The series of DB24C8-bearing copolymers obtained using various well-controlled feed ratios is summarized in Table 1.

### 3.2. Cloud Points Analysis of DB24C8-Bearing Copolymers

**Figure 1:**
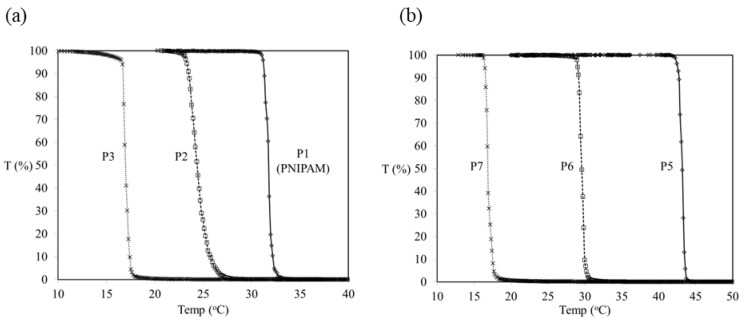
Transmittance of copolymers (0.5 wt %) in water.

The cloud points of copolymers in water were determined by turbidity experiments conducted at various temperatures. Figure 1 shows the change in transmittance of the synthesized copolymer solutions with temperature. The cloud point of each copolymer changed sharply following the incorporation of a small amount of DB24C8 monomer. For example, the introduction of 2.9% and 4.2% DB24C8 monomer, generating **P2** and **P3**, resulted in the cloud point decreasing from 31.1 °C (**P1**: PNIPAM) to 23.0 °C (**P2**) and 16.5 °C (**P3**), respectively (Figure 1a). The introduction of 15% AAM as a hydrophilic co-monomer resulted in an increase in the cloud point compared with in the absence of AAM. An increase in DB24C8 monomer to 2.4% and 5.0% resulted in the cloud points of the **P6** and **P7** copolymers containing AAM decreasing from 42.4 °C (**P5**: without DB24C8) to 28.9 °C (**P6**) and 16.2 °C (**P7**), respectively (Figure 1b). The cloud point of **P8**, containing 7.6% DB24C8 monomer, was estimated to decrease to approximately 4–5 °C, as the sample did not solidify even upon refrigeration. The cloud point of **P9**, containing 10.5% DB24C8 monomer, is likely lower than that of **P8,** and could be around 0 °C as estimated by the observation of copolymer solution in an ice bath.

### 3.3. Cesium Ion Binding to the DB24C8-Bearing Copolymers

**Figure 2:**
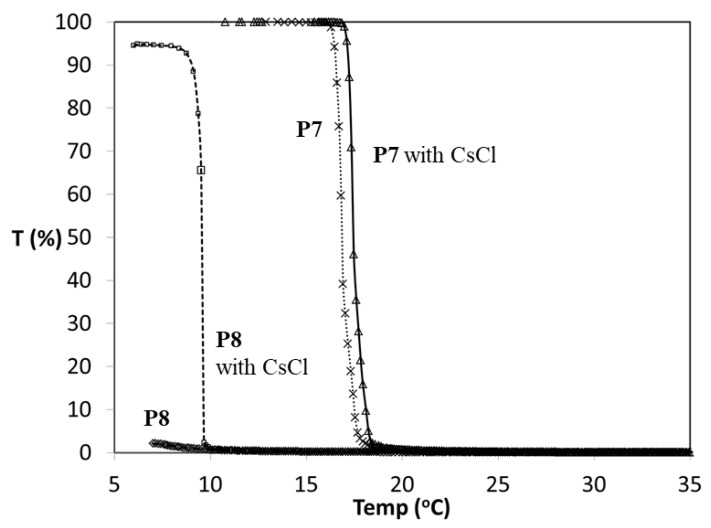
Transmittance of copolymers (0.5 wt %) in water in the absence/presence of CsCl.

We investigated the cloud points of **P7** and **P8** in aqueous solution with/without Cs^+^, and the results are shown in Table 2 and Figure 2. The cloud point of **P7** (DB24C8 = 5.0%) increased slightly (0.7 °C; 16.2→16.9 °C) in the presence of Cs^+^, whereas a larger cloud point shift (approximately 4–5 °C) was observed for **P8** (DB24C8 = 7.6%). The increase in cloud point in the presence of Cs^+^ may be due to repulsion between charged DB24C8-Cs^+^ complexes and an increase in hydrophilicity, as observed with PNIPAM bearing 18-crown-6 ether binding specific metal ions such as Pb^2+^ and Ba^2+^ [25]. An increase in DB24C8 monomer resulted in an increased cloud point shift, suggesting that the cloud point shift with **P9**, which contains a higher percentage of DB24C8 (10.5%), would be larger than that of **P7** in the presence of Cs^+^.

**Figure 3:**
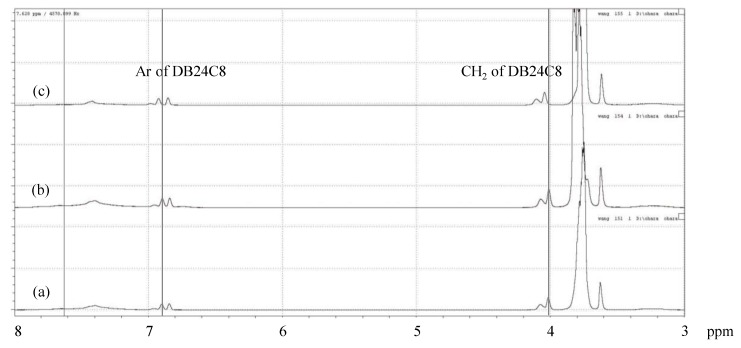
^1^H NMR spectra of **P7** in the presence of alkali metal salt: (**a**) None; (**b**) LiCl; (**c**) CsCl.

**Figure 4:**
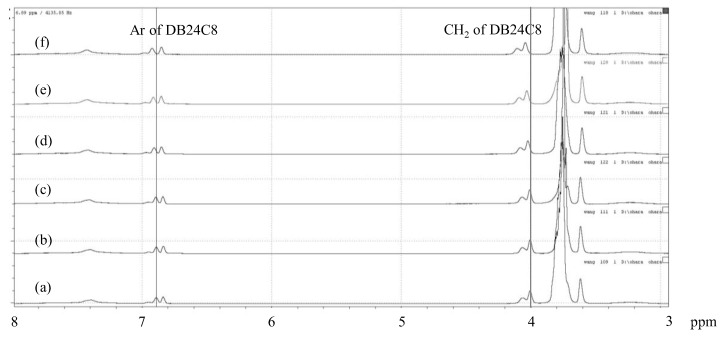
^1^H NMR spectra of **P9 **in the presence of alkali metal salt: (**a**) None; (**b**) LiCl; (**c**) NaCl; (**d**) KCl; (**e**) RbCl; (**f**) CsCl.

Next, we investigated the binding properties of copolymers bearing DB24C8 with various alkali metal ions using ^1^H NMR. The addition of Cs^+^ to copolymer solution resulted in a clear shift in the aromatic and methylene proton signals at 6.9 and 4.0 ppm, respectively, attributed to DB24C8 (Figure 3c and Figure 4f), compared to in the absence of alkali metal (Figure 3a and Figure 4a). No signal shift was observed following the addition of Li^+^ (Figure 3b and Figure 4b). The addition of the alkali metal ions K^+^, Rb^+^, and Cs^+^ resulted in gradual signal shifts of DB24C8, which increased with increasing ion size (Figure 4d–f; chemical shift changes (Δδ) are summarized in Table 3 and Table S1 (Supplementary Materials), suggesting that the binding affinity of copolymer bearing DB24C8 for alkali metal ion increases as the size of the ion increases. These trends of chemical shift change were similar to the results in the literature [7]. The Δδ of **P9** (Δδ = 0.037) was larger than that of **P7** (Δδ = 0.028) in the presence of Cs^+^, suggesting that the binding affinity for Cs^+^ of the higher DB24C8-content copolymer **P9 **(DB24C8 = 10.5%) was higher than that of the lower DB24C8-content copolymer **P7** (DB24C8 = 5.0%). Copolymer bearing DB24C8 **P9** exhibited the strongest binding for the large alkali metal ion Cs^+^ in the series of alkali metal ions tested in this study. The cavity diameter of DB24C8 is estimated to be about 4.5–5.0 Å [31,32], and is thus suitable for recognizing Cs^+^ (which has a diameter of 3.34 Å) [33]. Theoretically, **P9** can absorb 87 mg-Cs/g-copolymer if a crown ether molecule fully forms 1:1 binding with cesium ion.

Competition tests of Cs^+^ with other alkali metal ions were performed to investigate the binding selectivity of DB24C8 in the copolymer (Figures S12 and S13 (Supplementary Materials)). Table 4 shows the Δδ of methylene protons of DB24C8 in the copolymer **P9**. The addition of Cs^+^ to the copolymer solution in the presence of other Na^+^ and K^+^ ions resulted in clear shifts in the methylene proton signal at 4.0 ppm. Clear signal shifts were observed with high Na^+^ or K^+^ (CsCl:NaCl or KCl = 1:5). These results indicate that the binding affinity of DB24C8 in the copolymer is higher for Cs^+^ than that of Na^+^ and K^+^, suggesting that this adsorption system can remove Cs^+^ from an environment also including Na^+^ and K^+^.

## 4. Conclusions

Copolymers bearing DB24C8 were synthesized by radical copolymerization using a DB24C8-carrying AAM derivative and NIPAM as a monomer substrate. The cloud points of the copolymers decreased following the incorporation of a low content of hydrophobic DB24C8. The use of 15% hydrophilic AAM increased the cloud points of the DB24C8 copolymers. These copolymers bound alkali metal ions in aqueous solution, and the binding affinity was dependent on the size of the alkali metal ion. Our findings suggest that DB24C8-bearing polymers hold promise for adsorbing and removing cesium from the environment when used in conjunction with membrane separation including ultrafiltration, or with precipitation by centrifugation above the cloud point in which condition the NIPAM-based copolymers aggregate.

## Figures and Tables

**Table 1:** Synthesis of copolymers bearing DB24C8.

Polymer	Feed Molar Ratio of NIPAM/4/AAM	Conv (%) ^a^	Yield (%) ^b^	*M*_w _^c^ (×10^−3^)	*M*_w_/*M*_n _^c^	Molar Ratio of NIPAM/4/AAM in Polymer ^a^
**P1**	100/0/0	97	94	167	2.87	100/0/0
**P2**	97.0/3.0/0	92	95	248	1.62	97.1/2.9/0
**P3**	95.0/5.0/0	82	63	158	2.43	95.8/4.2/0
**P4**	93.5/6.5/0	95	76	210	2.02	93.7/6.3/0
**P5**	85.0/0/15	97	89	166	2.93	83.3/0/16.7
**P6**	82.0/3.0/15	98	80	244	1.54	85.7/2.4/11.9
**P7**	80.0/5.0/15	87	94	197	2.00	85.6/5.0/9.4
**P8**	77.5/7.5/15	98	84	176	2.95	81.7/7.6/10.7
**P9**	75.0/10/15	85	93	127	2.47	82.6/10.5/6.9

^a^ Determined by ^1^H NMR; ^b^ Isolated yield; ^c^ Determined by GPC.

**Table 2:** Cloud point values of **P7**, **P8**, and **P9** with/without Cs+ in water.

Polymer	Without Cs^+^ (°C)	With Cs^+^ (°C)
**P7**	16.2	16.9
**P8**	4–5 ^a^	9.2
**P9**	−3–0 ^b^	0–1 ^b^

^a^ Estimated by the observation of copolymer solution in a refrigerator (approximately 4 °C); ^b^ Estimated by the observation of copolymer solution in an ice bath.

**Table 3:** Chemical shifts (δ) and their changes (Δδ) of methylene protons of DB24C8 in **P9** in the presence of alkali metal salt.

	None	Li^+^	Na^+^	K^+^	Rb^+^	Cs^+^
Cation diameter (Å) [33]	-	1.20	1.90	2.66	2.96	3.34
δ (ppm)	4.005	4.006	4.008	4.022	4.030	4.042
Δδ (ppm)	-	0.001	0.003	0.017	0.025	0.037

**Table 4:** Chemical shifts (δ) and their changes (Δδ) of methylene protons of DB24C8 in **P9** in the competition test.

	None	NaCl	KCl	CsCl	CsCl + NaCl (CsCl:NaCl = 1:1)	CsCl + NaCl (CsCl:NaCl = 1:5)	CsCl + KCl (CsCl:KCl = 1:1)	CsCl + KCl (CsCl:KCl = 1:5)
δ (ppm)	4.005	4.008	4.022	4.042	4.043	4.040	4.047	4.054
Δδ (ppm)	-	0.003	0.017	0.037	0.038	0.035	0.042	0.049

## Supplementary Materials

The following are available online at http://www.mdpi.com/2073-4360/10/10/1095/s1, Figures S1–S11: NMR spectra of **4** and **P1**–**P9**, Figures S12 and 13: competition tests using **P9** by ^1^H NMR analysis, Table S1: Chemical shifts (δ) and their changes (Δδ) of methylene protons of DB24C8 in **P7** in the presence of alkali metal salt.
