# Supplementary material for: Synthesis and Characterization of Alkali Metal Ion-Binding Copolymers Bearing Dibenzo-24-crown-8 Ether Moieties
Source: Polymers (Basel). 2018 Oct 2;10(10):1095. doi: 10.3390/polym10101095 (PMC6403618; doi:10.3390/polym10101095)
Supplement: Supplementary file 1 [file polymers-10-01095-s001.pdf]

Supporting Information

Synthesis and Characterization of Alkali Metal Ion-Binding Copolymers Bearing Dibenzo-24-crown-8 Ether Moieties

Da-Ming Wang, Yuji Aso, Hitomi Ohara and Tomonari Tanaka *

Department of Biobased Materials Science, Graduate School of Science and Technology, Kyoto Institute of Technology, Kyoto 606-8585, Japan; d6861002@edu.kit.ac.jp (D.M.W.);
aso@kit.ac.jp (Y.A.); ohara@kit.ac.jp (H.O.)

* Correspondence: t-tanaka@kit.ac.jp; Tel.: +81-75-724-7802

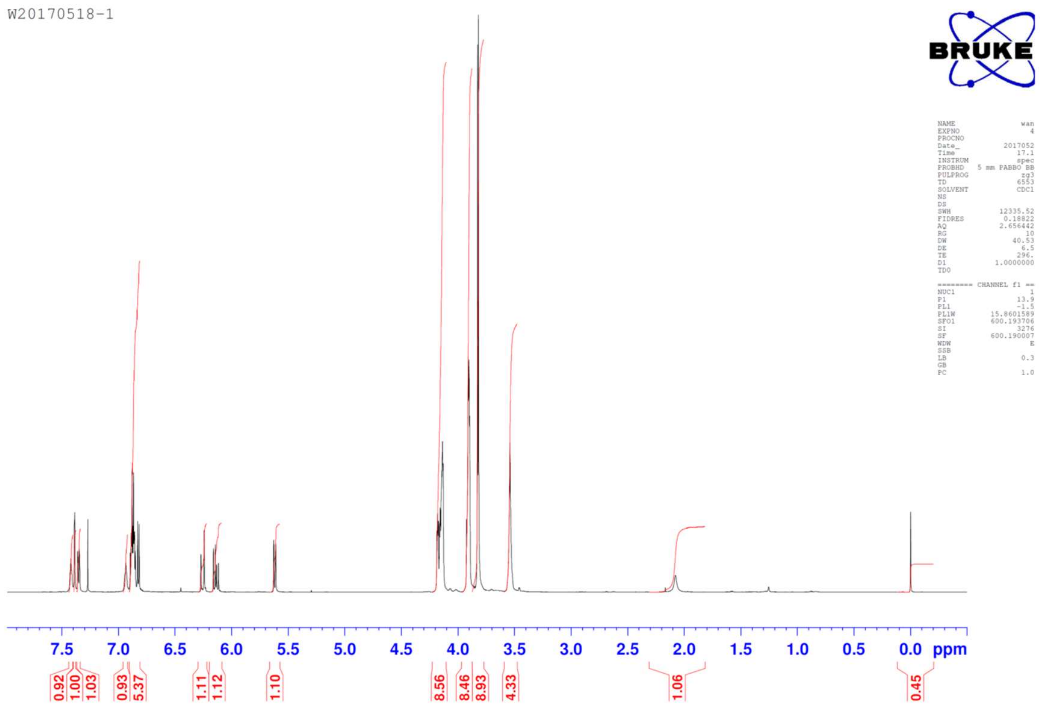

Figure S1. ^{1}H NMR spectrum of **4** in $CDCl_3$

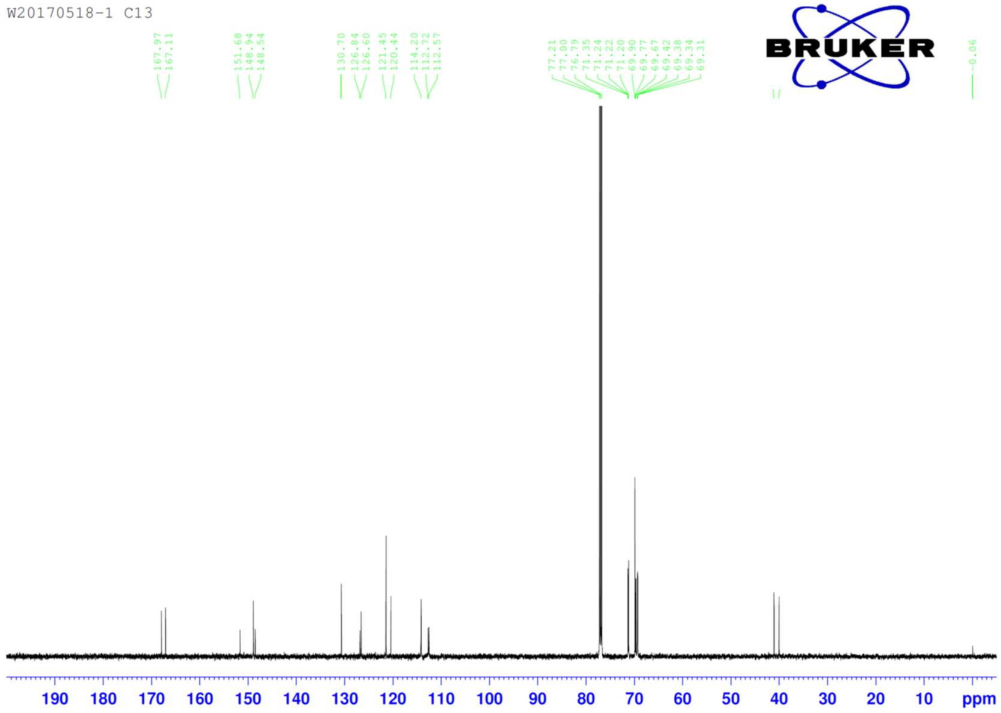

Figure S2. ^{13}C NMR spectrum of **4** in $CDCl_3$

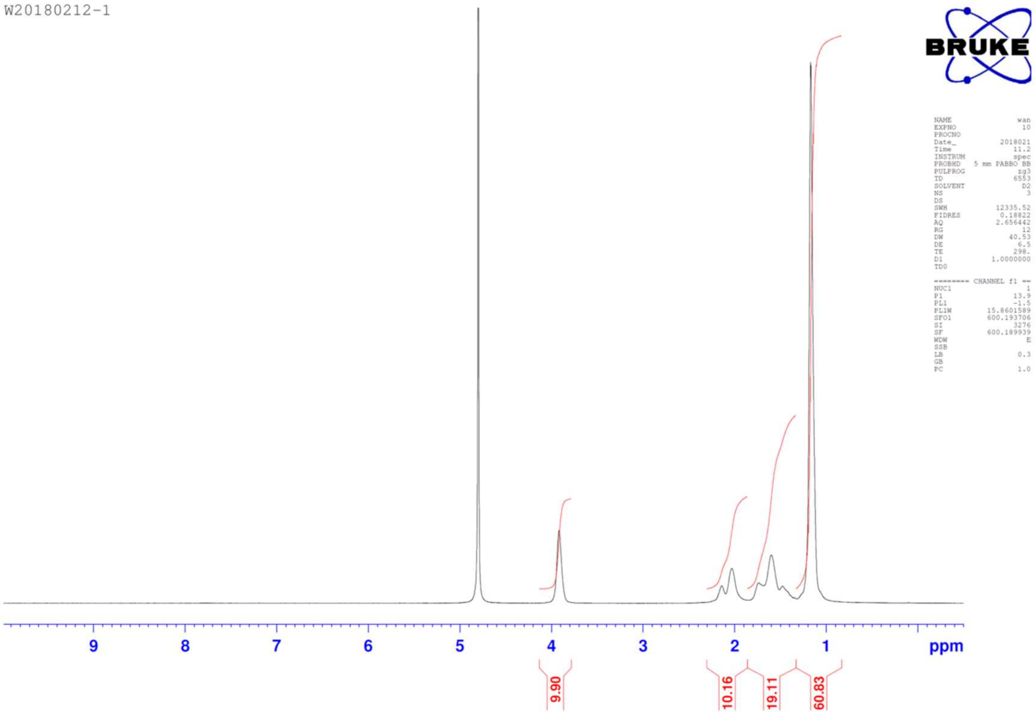

Figure S3. ^{1}H NMR spectrum of **P1** in D_2O

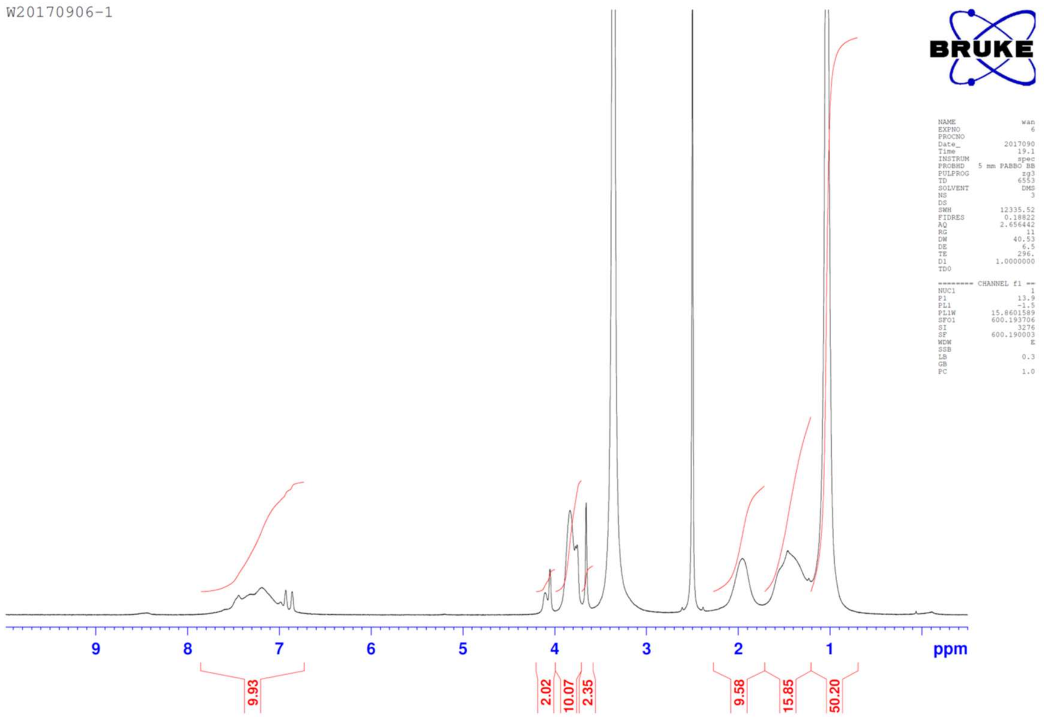

Figure S4. ^{1}H NMR spectrum of **P2** in DMSO-d_6

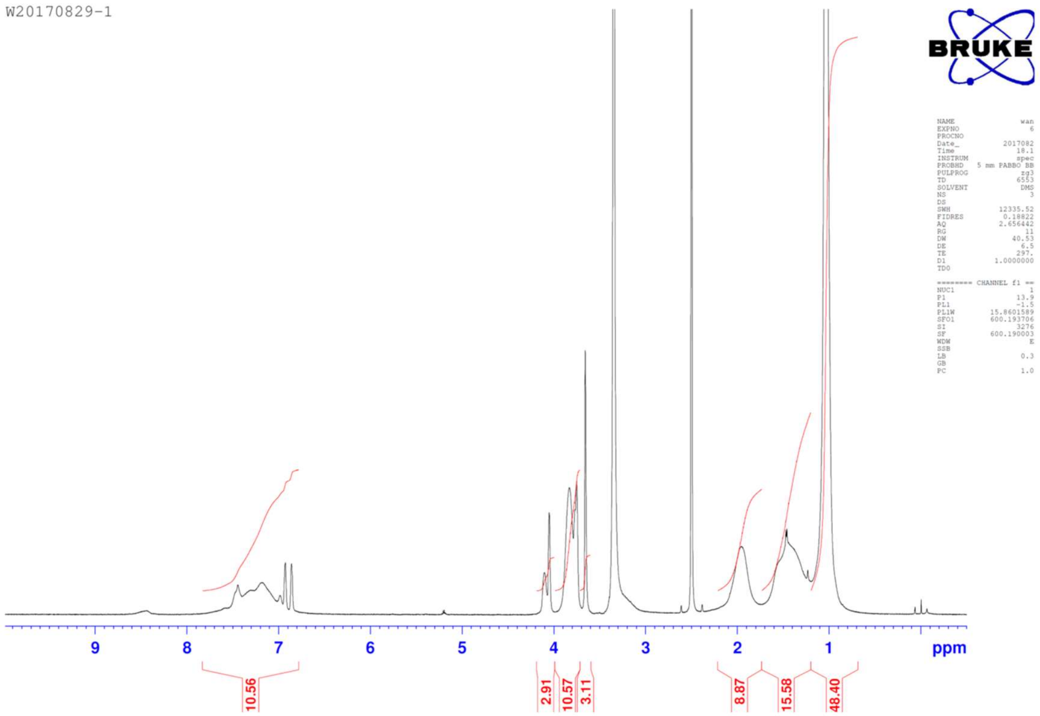

Figure S5. ^{1}H NMR spectrum of **P3** in DMSO-d_6

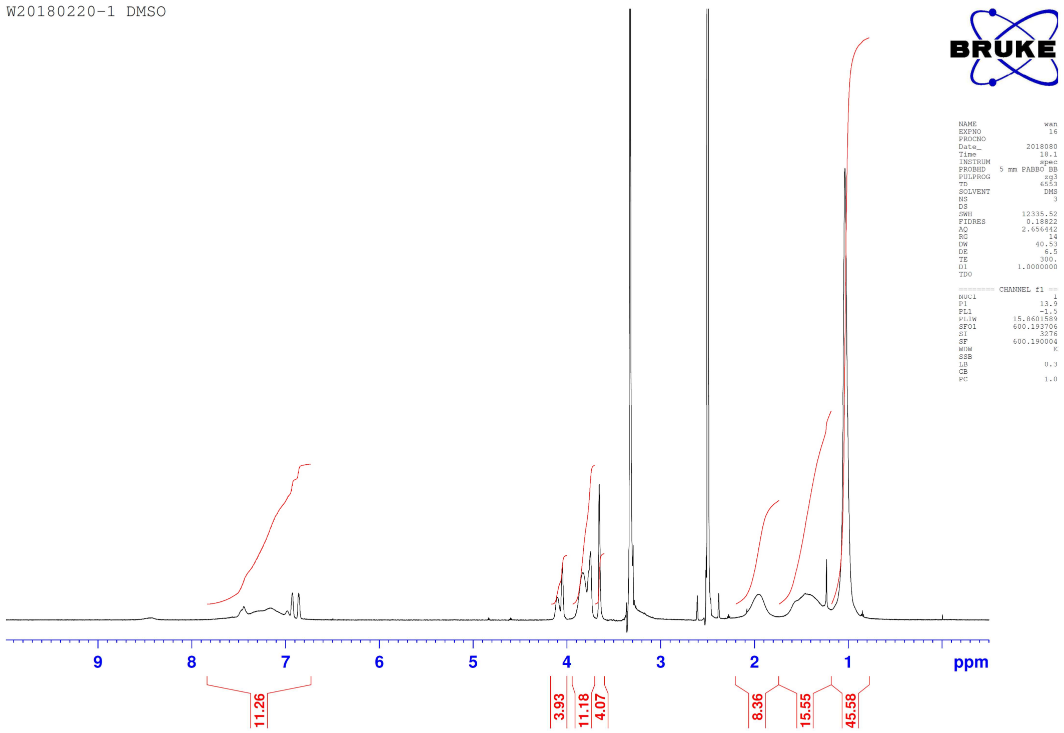

Figure S6. ^{1}H NMR spectrum of **P4** in DMSO-d_6

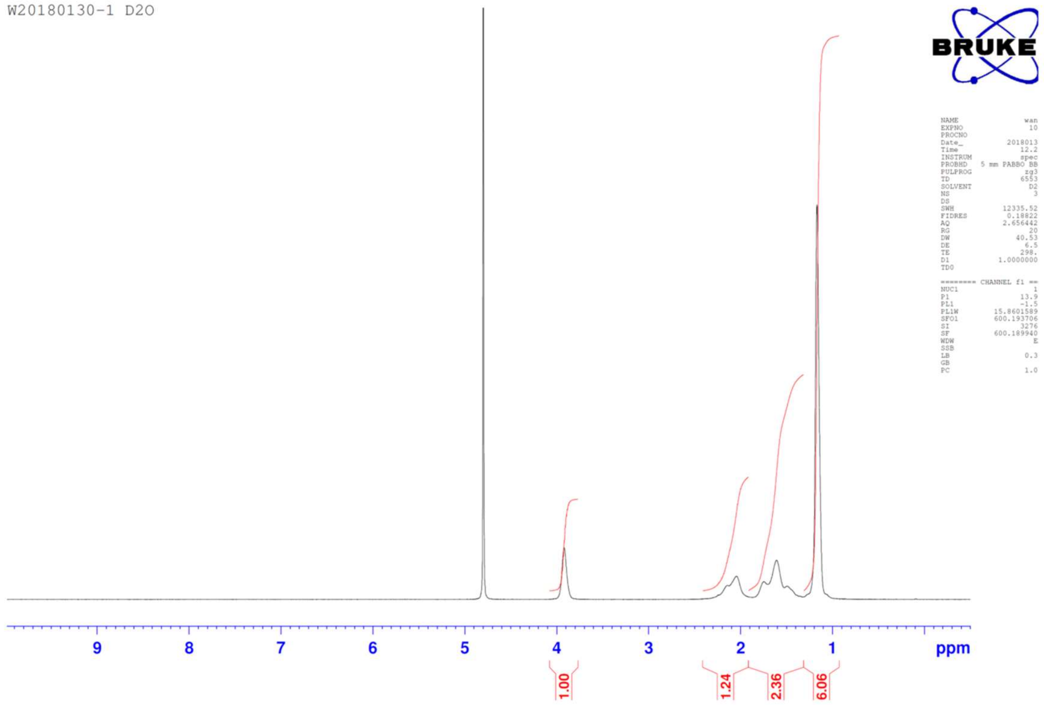

Figure S7. ^{1}H NMR spectrum of **P5** in D_2O

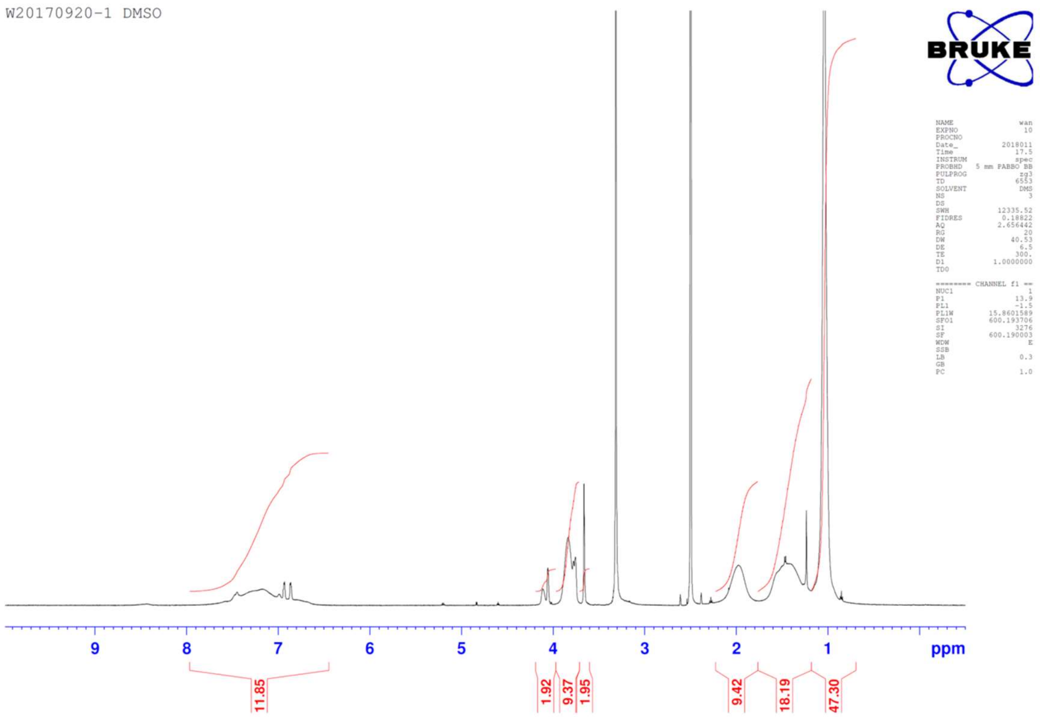

Figure S8. ^{1}H NMR spectrum of **P6** in DMSO-d_6

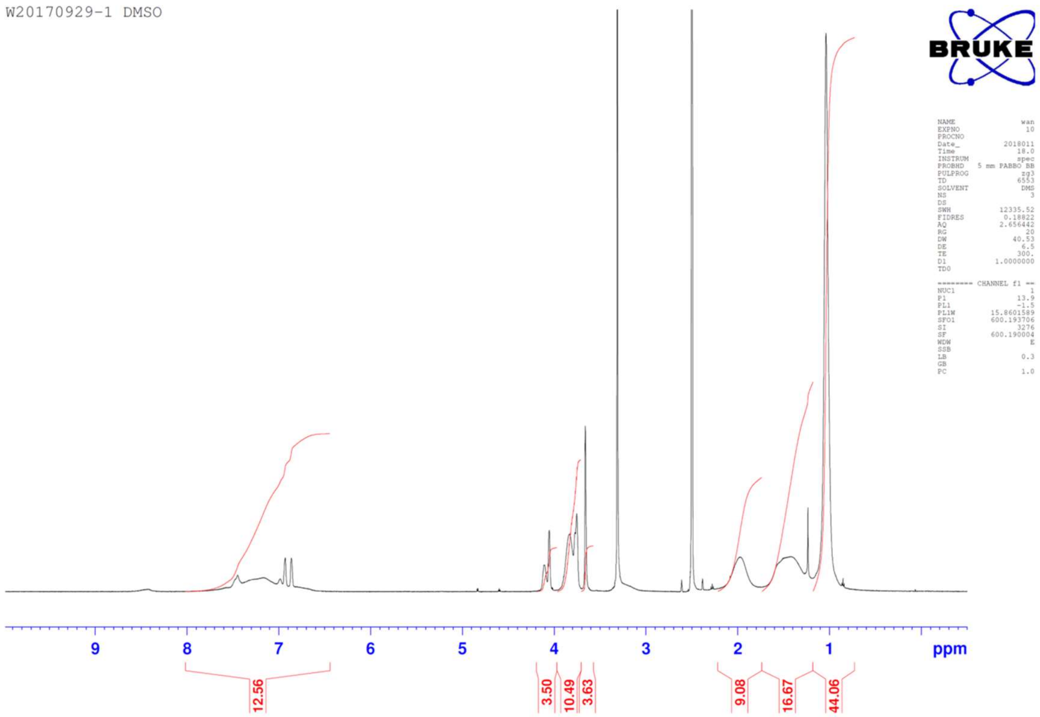

Figure S9. ^{1}H NMR spectrum of **P7** in DMSO-d_6

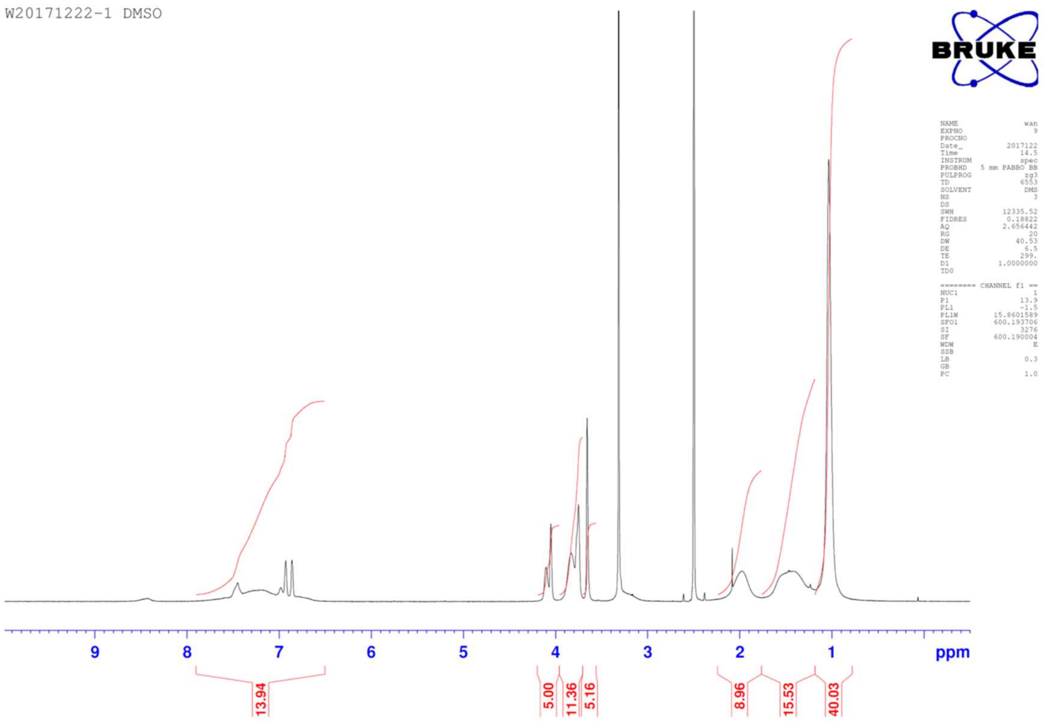

Figure S10. ^{1}H NMR spectrum of **P8** in DMSO-d_6

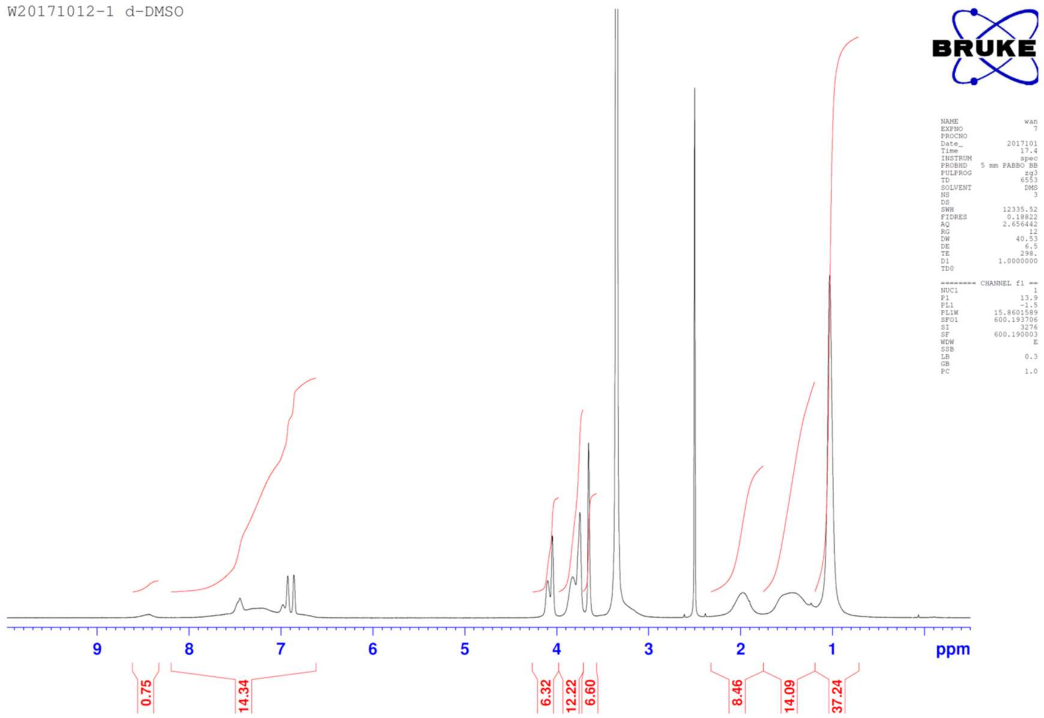

Figure S11. ^{1}H NMR spectrum of **P9** in DMSO-d_6

Table S1. Chemical shifts (δ) and their changes (Δδ) of methylene protons of DB24C8 in **P7** in the presence of alkali metal salt.

	none	Li^+	Cs^+
Cation diameter (Å) [33]	-	1.20	3.34
δ (ppm)	4.014	4.009	4.042
Δδ (ppm)	-	-0.005	0.028

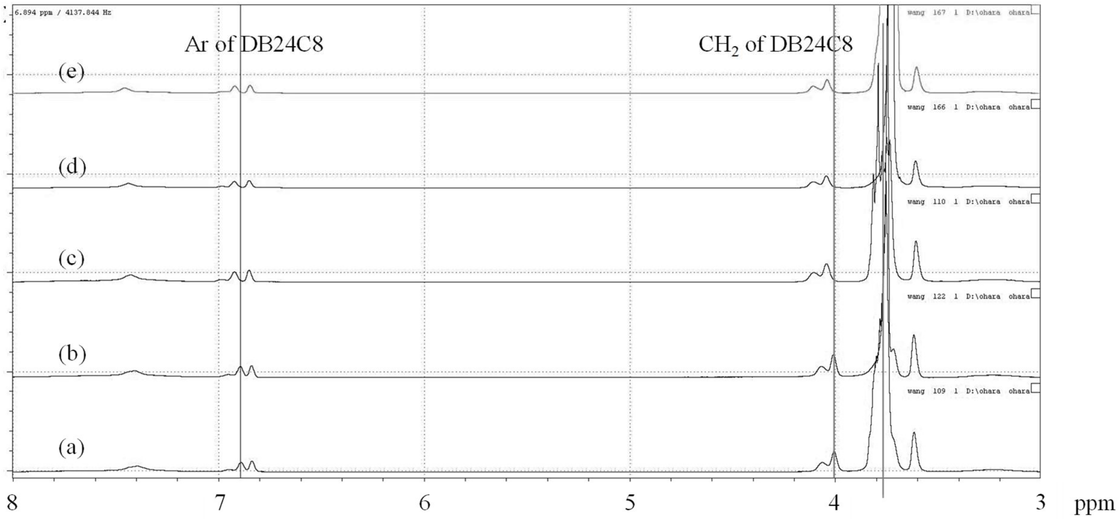

Figure S12. ^{1}H NMR spectra of **P9** in the presence of guest cations (DMSO-*d6*:D_2O = 7.5:1 v/v). (a) none; (b) NaCl; (c) CsCl; (d) CsCl + NaCl (CsCl:NaCl = 1:1); (e) CsCl + NaCl, (CsCl:NaCl = 1:5).

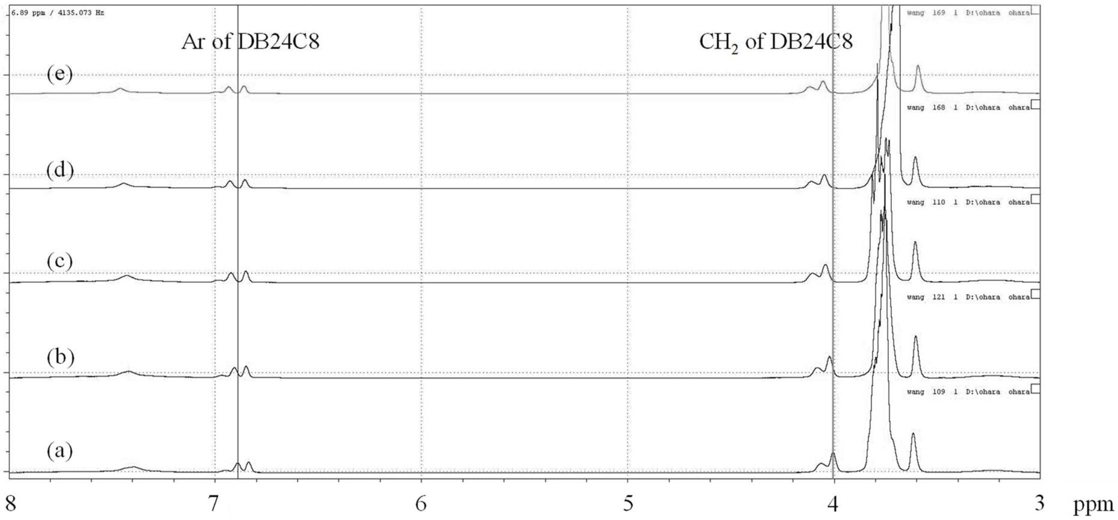

Figure S13. ^{1}H NMR spectra of **P9** in the presence of guest cations (DMSO-*d6*:D_2O = 7.5:1 v/v). (a) none; (b) KCl; (c) CsCl; (d) CsCl + KCl (CsCl:KCl = 1:1); (e) CsCl + KCl (CsCl:KCl = 1:5).
